# Expression of Concern: miR-26a prevents neural stem cells from apoptosis via β-catenin signaling pathway in cardiac arrest-induced brain damage

**DOI:** 10.1042/BSR-20181635_EOC

**Published:** 2020-07-02

**Keywords:** β-catenin, GSK-3β, miR-26a, NSCs and CA

The Editorial Office has been made aware of potential issues surrounding the scientific validity of this paper, hence has issued an expression of concern to notify readers whilst the Editorial Office investigates.

